# Cardiorespiratory fitness is associated with cerebral oxygen extraction in Alzheimer’s disease

**DOI:** 10.3389/fnagi.2026.1943472

**Published:** 2026-10-01

**Authors:** Paul J. Kueck, Zachary D. Green, Raechel Camones, Casey S. John, Robyn A. Honea, Jeffrey M. Burns, Eric D. Vidoni, Jill K. Morris

**Affiliations:** 1 University of Kansas Alzheimer’s Disease Research Center, Fairway, KS, United States; 2 Department of Neurology, University of Kansas Medical Center, Kansas City, KS, United States

**Keywords:** aging, Alzheimer’s disease, cardiorespiratory fitness, cerebral blood flow, oxygen extraction fraction

## Abstract

Cardiorespiratory fitness (CRF) is a modifiable determinant of brain health and has been associated with greater brain volume, improved cognition, and reduced dementia risk. However, whether CRF influences cerebral oxygen extraction fraction (OEF), a marker reflecting the balance between cerebral oxygen delivery and metabolic demand, remains poorly understood in aging and Alzheimer’s disease (AD). We performed a secondary analysis of the Acute Exercise Response on Brain Imaging and Cognition (AEROBIC) study to investigate relationships among CRF, cerebral blood flow (CBF), and OEF in cognitively healthy older adults (CH) and individuals with AD. Multivariable linear regression demonstrated that higher CRF was independently associated with lower OEF. A significant CRF × diagnosis interaction indicated that this relationship was present in AD but not CH. Across the full cohort, higher CBF was strongly associated with lower OEF, and this relationship was preserved across diagnostic groups. Regional analyses demonstrated similar inverse associations between CBF and OEF in multiple cortical regions involved in cognitive and autonomic regulation. These findings suggest that greater CRF may reduce reliance on oxygen extraction by improving the adequacy of cerebral oxygen delivery in AD. Together, CRF, CBF, and OEF provide complementary physiological information regarding cerebral oxygen metabolism and may improve understanding of cerebrovascular physiology in aging and AD.

## 1 Introduction

Alzheimer’s disease (AD) is the leading cause of dementia and is characterized by progressive cognitive decline and neuropathological hallmarks such as amyloid-beta plaque (Aβ) and neurofibrillary tangles (De-Paula et al., 2012; Rajmohan and Reddy, 2017). In addition to these pathological features, AD is associated with physiological alterations including diminished cerebral blood flow (CBF) (Spilt et al., 2002; Spilt et al., 2005; Weijs et al., 2023) and cerebrovascular dysfunction that may compromise the brain’s ability to adequately deliver oxygen to metabolically active tissue (Landau et al., 2011; Patel et al., 2024).

Prior studies have linked cardiovascular health to brain structure and function (Gonzalez et al., 2018; Kulshreshtha et al., 2019; Santos et al., 2017; Thacker et al., 2014). One important factor is cardiorespiratory fitness (CRF), an integrated measure of the ability of cardiovascular, respiratory, and muscular systems to deliver and utilize oxygen during sustained physical activity. CRF is typically indexed by peak oxygen uptake (VO2), particularly in older adults. Higher CRF has been associated with greater brain volume (Firth et al., 2018; Olivo et al., 2021; Plini et al., 2024), better cognitive function (Fiatarone Singh et al., 2014; Northey et al., 2018; Zhang et al., 2022), and a reduced risk of developing dementia (Hamer and Chida, 2009; Iso-Markku et al., 2022). These benefits are thought to arise, in part, through improvements in glycemic control (Shah et al., 2021), lower resting blood pressure (Stewart et al., 2005), and enhanced cerebral perfusion (Tomoto et al., 2023; van Hout et al., 2025), and other physiological adaptations that support neuronal metabolism and preserve structural brain integrity.

Cerebral oxygen metabolism depends on the coordinated interaction between systemic oxygen delivery, cerebral perfusion, and oxygen extraction. While peripheral measures characterize systemic fitness, neuroimaging approaches provide complementary measures of cerebral oxygen delivery and utilization and are needed to assess the cerebral perfusion and metabolic deficits observed in AD. Pseudo-continuous arterial spin labeling (pCASL) is a magnetic resonance imaging (MRI) technique to measure CBF and has shown reductions in individuals with AD compared to cognitively healthy older adults (CH) (Griffiths et al., 2001). T2-Relaxation-Under-Spin-Tagging (TRUST) MRI estimates venous blood oxygen saturation in the superior sagittal sinus (Lu and Ge, 2008; Lu et al., 2012; O'Brien et al., 2019). TRUST provides a non-invasive method for assessing oxygen extraction fraction (OEF) by coupling venous oxygen saturation with arterial blood oxygen saturation. OEF represents the proportion of delivered oxygen extracted from arterial blood by the brain and reflects the balance between cerebral oxygen delivery and metabolic demand. Accordingly, lower OEF indicates that metabolic requirements are met with less fractional oxygen extraction, whereas higher OEF reflects greater reliance on oxygen extraction to maintain cerebral oxygen metabolism (Jiang et al., 2020a; Jiang and Lu, 2022; Powers, 1991). CBF and OEF are often inversely related where reduced perfusion is often compensated by greater oxygen extraction (Peng et al., 2014). Given evidence that higher CRF supports cerebrovascular function and perfusion, CRF may influence cerebral metabolism through its effects on CBF and OEF.

Despite these advances, the relationship between CRF, OEF, and CBF in aging and AD remains poorly understood. Preliminary evidence in young healthy athletes suggests that greater CRF is associated with lower OEF (Bao et al., 2019). This may reflect a reduced reliance on oxygen extraction due to more adequate oxygen delivery. In older adults, lower OEF has also been associated with greater cognitive and amyloid burden, whereas higher OEF has been observed in those with greater vascular risk factors, suggesting complex alterations in cerebral oxygen metabolism across aging and disease states (Jiang et al., 2020b). However, the relationship between CRF and OEF in CH and AD remains unclear.

In this study, we conducted a secondary analysis of data from the Acute Exercise Response on Brain Imaging and Cognition (AEROBIC) study (ClinicalTrials.gov, ID: NCT04299308) to investigate the relationship between CRF and OEF in CH and AD groups. Higher CRF may enhance cerebral oxygen delivery through improvements in cardiovascular function and cerebral perfusion. Because OEF reflects the fraction of delivered oxygen that must be extracted to meet cerebral metabolic demand, more adequate oxygen delivery at rest would be expected to reduce OEF (Jiang and Lu, 2022). Thus, we hypothesized that individuals with higher CRF would demonstrate lower OEF. Because CBF and OEF are typically inversely related in healthy adults, we also examined whether this relationship is preserved in AD, where alterations in cerebrovascular and metabolic function may disrupt normal oxygen delivery-utilization coupling. By integrating measures of systemic fitness, cerebral perfusion, and oxygen extraction, this study aims to better characterize cerebral oxygen metabolism and determine how these physiological relationships are altered in AD.

## 2 Methods

### 2.1 Participants

The AEROBIC trial was a pilot randomized controlled trial to test the effect of acute exercise on brain glucose metabolism. The AEROBIC methods and primary outcomes have already been published (Green et al., 2024; Green et al., 2021). Briefly, participants were recruited from a volunteer registry at the University of Kansas Alzheimer’s Disease Research Center. Eligible participants were ≥60 years of age, on stable medications for ≥1 month, and were clinically classified at study enrollment as either CH [Clinical Dementia Rating (CDR) of 0] or having symptomatic AD (CDR 0.5 or 1) based on a standardized clinician diagnosis and remote CDR. Group assignment was therefore based on clinical status rather than biomarker classification, as the study was completed before plasma biomarkers were incorporated into routine diagnostic evaluation and before an FDA cleared AD biomarker test became available. Exclusion criteria have already been previously described (Green et al., 2021). All participants provided written consent on the day of their first visit after being fully informed of all procedures and risks associated with the study. This study was approved by the Institution Review Board at the University of Kansas Medical Center and performed in accordance with the standards set by the Declaration of Helsinki.

### 2.2 Procedure

The Graded Exercise Test (GXT) visit was scheduled in the morning following an overnight fast. The participant was connected to a 12-lead electrocardiograph and fitted for a mask to collect expired air (7,450 Series Silicone V2^™^ Oro-Nasal Mask, Hans Rudolph). An exercise physiologist familiarized each participant with the exercise equipment and explained the Borg Rating of Perceived Exertion (RPE). After 5 min of quiet rest, the participant remained seated, and baseline measurements were collected for 2 min. Our GXT method has been previously published (Billinger et al., 2011). To summarize, all participants began walking at a pace of 1.7 miles per hour at 0% grade. At 2-min intervals, the grade, speed, or both were increased. Blood pressure and RPE were acquired during the last 30 s of each interval. Expired gases were collected continuously, and oxygen uptake and carbon dioxide production were averaged at 30-s intervals (Parvomedics, Sandy, UT). Peak VO2 was defined as the highest 30-s oxygen uptake value achieved during the test. The exercise test was terminated if the participant reached volitional exhaustion or met 3 of 4 criteria; RPE > 17, RER > 1.1, Plateau in VO_2_, HR > 90% age predicted max (Howley et al., 1995; Midgley et al., 2007). CRF was assessed as peak VO2 before volitional exhaustion or symtptom-limited termination.

### 2.3 Blood collection and processing

Blood samples were collected using EDTA as an anti-coagulant at rest following an overnight fast. Blood was centrifuged at 1500 x g for 10 min to generate plasma, which was immediately frozen. EDTA plasma was used to analyze phosphorylated Tau 217 (pTau217) using a Simoa HD-X (Quanterix, Billerica, MA). Aβ 40 and 42 were assessed using a Lumipulse G1200 (Fujirebio, Malvern, PA). Values were used as a ratio (Aβ42:40), where lower values reflect greater Aβ burden. Whole blood was collected using ACD as an anti-coagulant and used to genotype apolipoprotein E polymorphisms (*APOE*) using TaqMan single nucleotide polymorphism allelic discrimination assay (Thermo Fisher, Waltham, MA). Participants were classified as an APOE4 carrier if they carried at least one APOE4 allele.

### 2.4 Neuroimage acquisition

On a separate day, participants returned for a resting MRI scan in a non-fasted state. MRI scans were performed using a 3 T Siemens Skyra scanner (Erlangen, Germany) with a 20-channel head/neck receiver coil.

A T1-weighted magnetization-prepared rapid gradient echo (MPRAGE) sequence was acquired for anatomical reference (TR/TE/TI = 2300/2.98/900 ms, flip angle = 9°, FOV = 256 × 240 mm^2^, 176 slices, slice thickness = 1 mm). CBF was measured using a background-suppressed pCASL sequence with a 3D GRASE readout (TR/TE = 4300/22.42 ms, flip angle = 120°, FOV = 300 × 300 mm^2^, matrix = 96 × 66, voxel size = 3.1 × 3.1 × 2.5 mm^3^, 48 slices, post-labeling delay = 2 s, four-segment acquisition without partial Fourier reconstruction, readout duration = 23.1 ms, total scan time = 5:48 min, two M0 images). Sagittal sinus venous oxygenation was assessed using TRUST sequence [(TR/TE = 3000/7.1 ms, flip angle = 90°, FOV = 64 × 64 mm^2^), total scan time = 48 s].

### 2.5 Neuroimage processing

All neuroimages underwent visual quality assessment by study personnel to identify excessive motion, image distortion, signal dropout or other acquisition-related artifacts. Only datasets deemed of acceptable quality were included in subsequent analyses. T1-weighted MPRAGE images were segmented into gray matter (GM), white matter (WM), and cerebrospinal fluid (CSF) using CAT12 (neuro.uni-jena.de/cat), which was also used to estimate total intracranial volume (TIV) (Gaser et al., 2024). Participant-specific anatomical regions of interest (ROI) were generated from the Neuromorphometrics atlas (Neuromorphometrics Inc. Somerville, MA, United States). Next, pCASL label and control images were motion corrected by realigning each image to the corresponding reference image acquired following the M0 image. Perfusion-weighted images were generated using surround subtraction of label-control pairs and averaged to produce a quantitative CBF map. CBF maps were then coregistered to the participant’s anatomical image and smoothed using a 6-mm full-width at half-maximum Gaussian kernel (Wang et al., 2008). Regional ROIs CBF were estimated using participant-specific GM probability maps derived from CAT12 segmentation. GM probability maps were thresholded to retain voxels with a GM probability >0.2 and intersected with the Neuromorphometrics atlas to generate individualized cortical GM mask. The resulting cortical GM mask was binarized, and mean CBF was extracted from each participant’s quantitative CBF map. This approach restricted CBF analyses to predominantly GM and minimized contributions from WM and CSF, however, formal partial volume correction was not performed.

CBF ROIs were categorized into three groups: (1) whole-brain cortical gray matter to provide a global index of perfusion; (2) regions corresponding to the Central Autonomic Network, including the anterior cingulate cortex, bilateral insula, thalamus, and brainstem which mediates cardiovascular and respiratory regulation; and (3) regions corresponding to the Default Mode Network, including the posterior cingulate cortex, precuneus, medial frontal cortex, parahippocampus, and hippocampus, a network consistently implicated in AD-related functional abnormalities. CBF ROIs were defined as *a priori* based on prior literature demonstrating their involvement in autonomic and cardiopulmonary regulation (Critchley et al., 2000; Motl et al., 2015; Yamanaka et al., 2025), and their susceptibility to AD–related pathology (Andrews-Hanna et al., 2010; Greicius et al., 2004).

For TRUST processing, transverse relaxation time of venous blood (T2b) and venous oxygenation (Yv) values in the superior sagittal sinus were estimated using a semi-automated pipeline based on a modified script to incorporate FMRIB Software Library (FSL) tools (Jenkinson et al., 2012; Smith et al., 2004; Woolrich et al., 2009). The TRUST data consisted of a single-slice 64 × 64 matrix, with different effective echo times (TE) concatenated in time.

Processing began by computing pairwise subtracted images, specifying the number of TE and applying control-tag differencing. The ROI was manually restricted to the superior sagittal sinus, and a binary mask was generated by thresholding at the 99th percentile signal intensity within the ROI, corresponding to the top ~1% of voxels. The resulting mask was used to extract a mean signal time course.

Subsequent quantification was performed in MATLAB using a custom script. The signal decay across the echo times was fitted to a single exponential model with TE of 0, 40, 80, and 160 ms. The fitted decay rate was used to compute T2b from the relationship assuming a blood T₁ of 1,624 ms. Yv was then calculated from T2b using a quadratic model which relates T2b to venous oxygen saturation based on assumed hematocrit to be 42% for males and 40% for females, resulting in the final values of T2b [in milliseconds (ms)] and Yv (in %) (Lu et al., 2012; Liu et al., 2016).

Arterial oxygenation (Ya) was assumed to be 98% consistent with prior studies. Based on the Fick principle, OEF was defined as the arteriovenous oxygen difference normalized to arterial oxygen content. Assuming oxygen content is proportional to hemoglobin saturation, OEF was calculated as (Ya – Yv)/Ya x 100 (Lin et al., 2019; Yan et al., 2026).

### 2.6 Statistical analyses

All statistical analyses were performed using IBM SPSS Statistics (Version 29.0). Participant characteristics were summarized as mean ± standard deviation (SD) or frequency (%), as appropriate. Demographic characteristics were compared between CH and AD groups using independent samples t-tests for continuous variables and chi-square tests for categorical variables.

GXT variables and baseline neuroimaging measures were compared between diagnostic groups using analysis of covariance (ANCOVA). GXT models were adjusted for age and sex, whereas neuroimaging models were additionally adjusted for TIV.

To evaluate the primary hypothesis that higher CRF would be associated with lower OEF, multivariable linear regression models were constructed with OEF as the dependent variable and relative CRF as the primary independent variable. All regression models were adjusted for age, sex, diagnosis, and TIV. To determine whether the association differed by diagnostic group, an interaction term (CRF × diagnosis; diagnosis coded as CH = 0 and AD = 1) was added to the adjusted model. Because the interaction was statistically significant, stratified regression analyses were subsequently performed separately within the CH and AD groups using the same covariates.

To evaluate the relationship between CBF and OEF, multivariable linear regression models were constructed with OEF as the dependent variable and CBF as the primary independent variable, adjusting for age, sex, diagnosis, and TIV. A CBF × diagnosis interaction term was subsequently added to assess whether this relationship differed between diagnostic groups.

Exploratory ROI analyses were performed in whole-brain cortical gray matter and predefined regions comprising the Central Autonomic Network and Default Mode Network. Ten ROI comparisons were performed, with each model adjusted for age, sex, diagnosis, and TIV. To account for multiple comparisons, a Bonferroni-corrected significance threshold was applied for ROI analyses (α = 0.05/10 = 0.005). Statistical significance for all other analyses was defined as *p* < 0.05.

## 3 Results

Participant characteristics are provided in Table 1. Due to a technical issue, the TRUST MRI sequence was added partway through the study; therefore, participants who did not receive this scan were excluded (*n* = 2). Participants who were unable to complete the full MRI (i.e., discomfort/anxiety) were also excluded (*n* = 7). Additionally, individuals with excessive motion artifacts were removed from analyses (*n* = 3). After these exclusions, the final sample consisted of 48 participants (24 CH and 24 AD). Across the cohort, the average time elapsed between the GXT and MRI visits was 34 days, and this interval did not differ significantly between groups (*p* = 0.651). The participants in the AD group were older (*p* < 0.001), and a larger percent carried the *APOE4* allele (*p* = 0.041) compared to the CH group. AD had a lower Aβ42:40 ratio (*p* < 0.001) and higher pTau217 (*p* < 0.001) compared to CH, reflecting greater Aβ burden. There were no group differences in number of females, education and BMI (*p* = 0.999, *p* = 0.567, *p* = 0.244, respectively).

**Table 1:** Participant demographics, graded exercise test assessment, and global neuroimaging outcomes between groups.

	Overall (*n* = 48)	Cognitively healthy (*n* = 24)	Alzheimer’s disease (*n* = 24)	*p*-value
^a^Demographics				
Age, years	72 [7]	68 [5]	76 [7]	**<0.001**
No. female [%]	20 [42]	10 [42]	10 [42]	0.999
Education, years	17 [2]	17 [2]	17 [3]	0.567
BMI, Kg/m^2^	25.9 [4]	26.6 [5]	25.1 [4]	0.244
No. *APOE4* carrier [%]	22 [46]	7 [29]	15 [63]	**0.041**
Aβ42:40 ratio	0.0936 [0.011]	0.1002 [0.009]	0.0870 [0.009]	**<0.001**
pTau217, pg./mL	0.599 [0.48]	0.267 [0.12]	0.932 [0.48]	**<0.001**
Time between visits, days	34 [11]	34 [10]	33 [11]	0.651
^b^Graded exercise test				
Baseline HR, bpm	64 [11]	63 [10]	65 [11]	0.718
Baseline MAP, mmHg	119 [14]	116 [10]	121 [16]	0.230
Baseline respiratory exchange ratio	0.75 [0.1]	0.77 [0.1]	0.75 [0.1]	0.518
Cardiorespiratory fitness, Relative peak VO2 (mL·kg^−1^·min^−1^)	24.7 [7]	26.9 [9]	22.6 [4]	0.544
Peak HR, bpm	153 [13]	160 [9]	146 [13]	**0.013**
90% predicted HR Achieved [%]	45 [94]	23 [96]	22 [92]	0.500
Peak MAP, mmHg	122 [15]	125 [16]	119 [13]	0.458
Peak respiratory exchange ratio	1.12 [0.1]	1.13 [0.1]	1.12 [0.1]	0.793
Peak RPE	19 [1]	19 [1]	18 [1]	0.642
Exercise duration, seconds	695 [185]	797 [158]	603 [160]	**0.006**
^c^Neuroimaging				
Total gray matter volume, mL	595 [74]	628 [66]	562 [68]	**<0.001**
Global CBF (ml/100 g/min)	60.3 [13]	65.5 [12]	55.2 [13]	**0.009**
OEF, percent	42 [8]	42 [8]	43 [8]	0.604

a Demographics analyses used chi-square tests were used for categorical variables and t-test for continuous variables.

b Graded Exercise Test variables were compared between diagnostic groups adjusting for age and sex.

c Neuroimaging analyses compared between diagnostic groups adjusting for age, sex, total intracranial volume.

E4, apolipoprotein E4 allele; pTau217, phosphorylated Tau217; Aβ42, amyloid-beta 42; BMI, body mass index; HR, heart rate; MAP, mean arterial pressure; VO2, peak oxygen uptake; RPE, rating of perceived exertion; CBF, cerebral blood flow; OEF, oxygen extraction fraction.

Baseline measures showed no difference in HR, MAP, and RER (*p* = 0.718, *p* = 0.230, *p* = 0.518, respectively) between groups. Early termination of GXT occurred in six participants due to fatigue (CH = 2 and AD = 3) and one participant due to safety concern. Following GXT, AD had a lower maximum HR achieved (*p* = 0.013) and duration (*p* = 0.006) compared to CH, however there was no difference in percent of participants who achieved 90% of the age predicted HR max between the two groups (*p* = 0.500). Furthermore, no difference in peak VO2, MAP, RER, and RPE (*p* = 0.544, *p* = 0.458, *p* = 0.793, *p* = 0.642, respectively) was observed between groups. Cross-sectional imaging analyses showed AD to have lower global GM volume (*p* < 0.001) and global GM CBF (*p* = 0.009) compared to CH, but no difference in OEF (*p* = 0.604) was observed between groups. Group differences in GXT and neuroimaging outcomes have been summarized in Table 1.

To determine whether the established inverse relationship between CBF and OEF was preserved in older adults with and without AD, linear regression was performed across the full cohort. In an unadjusted model, higher CBF was significantly associated with lower OEF (β = −0.488, *p* < 0.001) (Figure 1B). This association remained significant after adjustment for age, sex, diagnosis, and TIV (β = −0.667, *p* < 0.001). Diagnosis was also an independent predictor of OEF (*p* = 0.019). To determine whether the association between CBF and OEF differed by diagnostic group, a CBF × diagnosis interaction term was added to the adjusted model. The interaction was not statistically significant (β = −0.134, *p* = 0.485), indicating that the association between CBF and OEF did not differ by diagnostic group, Table 2.

**Figure 1:**
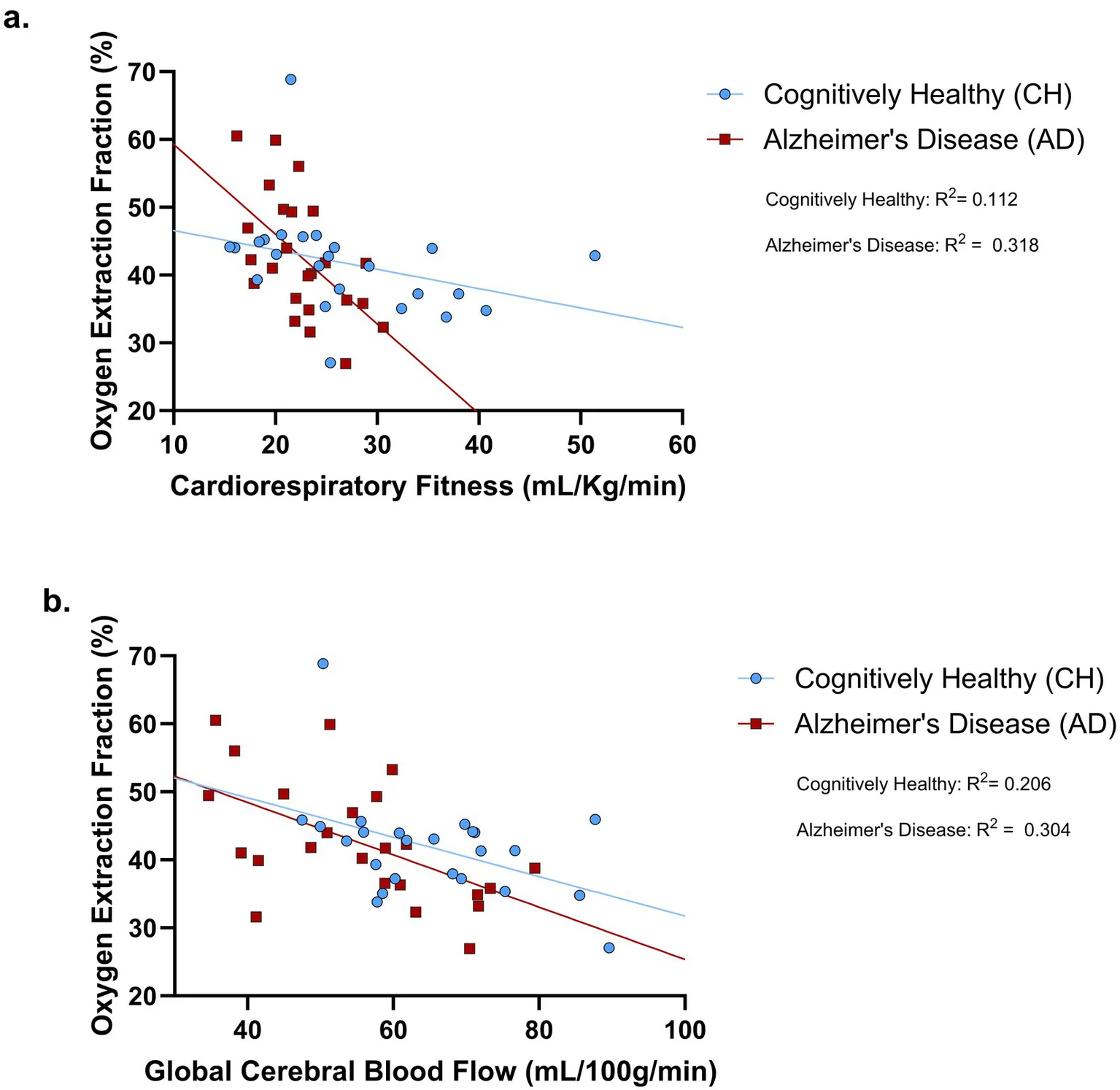
**(a)** Relationship between OEF and cardiorespiratory fitness. **(b)** Relationship between OEF and cerebral blood flow.

**Table 2:** Primary multivariable linear regression analyses examining the associations of cardiorespiratory fitness (CRF) and cerebral blood flow (CBF) with oxygen extraction fraction (OEF).

Analysis	Predictor	B (95% CI)	Standardized β	*p*-value
Adjusted model	CRF	**−0.451 (−0.865, −0.036)**	**−0.388**	**0.034**
Interaction model	CRF × Diagnosis	**−1.049 (−2.010, −0.088)**	**−0.369**	**0.033**
Stratified (CH)	CRF	−0.274 (−0.786, 0.239)	−0.320	0.277
Stratified (AD)	CRF	**−1.448 (−2.511, −0.385)**	**−0.616**	**0.010**
Adjusted model	Global CBF	**−0.413 (−0.585, −0.240)**	**−0.667**	**<0.001**
Interaction model	CBF × Diagnosis	−0.117 (−0.453, 0.219)	−0.134	0.485

OEF was the dependent variable in all models. Adjusted models included age, sex, diagnosis, and total intracranial volume (TIV) as covariates. Interaction models additionally included the CRF × diagnosis or CBF × diagnosis term to assess whether these associations differed by diagnostic group. Because the CRF × diagnosis interaction was statistically significant, stratified analyses were subsequently performed separately within the cognitively healthy (CH) and Alzheimer’s disease (AD) groups using the same covariates, excluding diagnosis. Diagnosis was coded as CH = 0 and AD = 1. Unstandardized regression coefficients (B), 95% confidence intervals (CI), standardized beta coefficients (β), and p-values are presented. Bold values indicate statistical significance defined as *p* < 0.05.

Regional analyses were conducted to examine the regional pattern of the association between CBF and global OEF across the full cohort. Because TRUST-derived OEF represents global measure of cerebral oxygen extraction rather than regional estimate, these analyses should be interpreted as associations between regional perfusion and whole-brain oxygen extraction. Across multiple brain regions, higher CBF was consistently associated with lower OEF, Table 3. The strongest inverse associations was observed in anterior cingulate cortex (β = −0.552, *p* < 0.001), insula (β = −0.532, *p* < 0.001), thalamus (β = −0.517, *p* = 0.002), posterior cingulate cortex (β = −0.577, *p* < 0.001), precuneus (β = −0.600, *p* < 0.001), and medial frontal cortex (β = −0.586, *p* < 0.001). No significant association was observed in the brainstem, hippocampus, and parahippocampal gyrus. All significant regional associations remained significant after Bonferroni correction for multiple comparisons (α = 0.005).

**Table 3:** Regional multivariable linear regression analyses examining the associations between cerebral blood flow (CBF) and oxygen extraction fraction (OEF) across the full cohort.

Region	B (95% CI)	Standardized β	Adjusted R^2^	*p*-value
Global				
Cortical gray matter	**−0.413 (−0.585, −0.240)**	**−0.667**	**0.318**	**<0.001**
Central autonomic network				
Anterior cingulate cortex	**−0.353 (−0.540, −0.166)**	**−0.552**	**0.214**	**<0.001**
Insula	**−0.432 (−0.673, −0.192)**	**−0.532**	**0.194**	**<0.001**
Thalamus	**−0.306 (−0.493, −0.119)**	**−0.517**	**0.159**	**0.002**
Brainstem	−0.445 (−0.794, −0.095)	−0.492	0.085	0.014
Default mode network				
Posterior cingulate cortex	**−0.250 (−0.390, −0.109)**	**−0.577**	**0.189**	**<0.001**
Precuneus	**−0.234 (−0.355, −0.112)**	**−0.600**	**0.220**	**<0.001**
Medial frontal cortex	**−0.376 (−0.568, −0.185)**	**−0.586**	**0.229**	**<0.001**
Parahippocampal gyrus	−0.279 (−0.629, 0.071)	−0.279	0.003	0.115
Hippocampus	−0.279 (−0.565, 0.007)	−0.330	0.031	0.056

OEF was the dependent variable and regional CBF was the primary predictor in all models. All models were adjusted for age, sex, diagnosis, and total intracranial volume (TIV). Regions are grouped into global cortical gray matter, the Central Autonomic Network, and the Default Mode Network. Unstandardized regression coefficients (B), 95% confidence intervals (CI), standardized beta coefficients (β), adjusted R^2^ values, and *p*-values are presented. Statistical significance for the regional analyses was determined using a Bonferroni-corrected threshold of *p* < 0.005 (0.05/10). Bold p-values indicate associations that remained significant after Bonferroni correction.

To test our first hypothesis that higher CRF will demonstrate reduced OEF, linear regression was conducted. In an unadjusted model, CRF was significantly associated with OEF (β = −0.426, *p* = 0.010). After adjustment, CRF remained a significant independent predictor of OEF (β = −0.388, *p* = 0.034).

To formally test whether diagnosis moderated this relationship, a CRF × diagnosis interaction term was added to the adjusted multivariable regression model. The interaction was significant (β = −0.369, *p* = 0.033), indicating that the association between CRF and OEF differed by diagnostic group (Figure 1A). Follow-up stratified analyses demonstrated a significant inverse association between CRF and OEF in the AD group (β = −0.616, *p* = 0.010), whereas no significant association was observed in the CH group (β = −0.320, *p* = 0.277) (Table 2).

## 4 Discussion

The present findings support a model in which systemic cardiorespiratory fitness, cerebral perfusion, and oxygen extraction represent complementary components of cerebral oxygen delivery and utilization. Consistent with our first hypothesis, higher CRF was associated with lower OEF. Notably, this relationship differed across diagnostic groups. In individuals with AD, higher relative CRF was strongly associated with lower OEF, whereas statistical significance was no reached in CH. Because OEF reflects the fraction of delivered oxygen that must be extracted to satisfy cerebral metabolic demand (Jiang et al., 2020a; Jiang and Lu, 2022; Powers, 1991), these findings suggest that greater cardiorespiratory fitness is associated with reduced reliance on oxygen extraction, potentially reflecting differences in cerebral oxygen delivery, utilization or both. An alternative, although not mutually exclusive, explanation is that individuals with higher CRF may exhibit more efficient cellular oxygen utilization, thereby requiring less fractional oxygen extraction to support cerebral metabolic demands. Thus, lower OEF could reflect improved metabolic efficiency rather than enhanced oxygen delivery alone. Because cellular metabolic function was not directly measured in the present study, the relative contributions of these mechanisms cannot be determined.

The significant CRF × diagnosis interaction suggests that the relationship between CRF and cerebral oxygen extraction differs by diagnostic status and may become more apparent in the presence of AD-related cerebrovascular and metabolic dysfunction. Importantly, this finding was observed despite the absence of group differences in either CRF or OEF. Thus, the present results should not be interpreted as reflecting differences in average fitness or oxygen extraction between groups, but rather differences in how CRF relates to cerebral oxygen extraction. Specifically, higher CRF was associated with lower OEF in AD, whereas the association did not reach statistical significance in CH, suggesting that AD may alter the association between systemic fitness and cerebral oxygen metabolism.

One possible explanation is that individuals with AD often exhibit both cerebrovascular damage (Iadecola and Gottesman, 2018; Love and Miners, 2016) and metabolic dysfunction (Swerdlow, 2020; Yin et al., 2016), which may narrow the margin between oxygen delivery and metabolic demand. Under these conditions, relatively small differences in systemic CRF may have a greater influence on cerebral oxygen delivery, thereby reducing the OEF required to maintain resting metabolism.

Cerebral metabolic abnormalities in AD may further heighten this sensitivity. Impaired brain glucose metabolism (de Leon et al., 2001; Phelps and Mazziotta, 1985) and mitochondrial dysfunction (Swerdlow, 2020; Swerdlow, 2018). are well established and at the cellular level, AD is associated with reductions in mitochondrial number and size (Baloyannis, 2006; Brown et al., 2001; de la Monte et al., 2000; Hirai et al., 2001). These changes can limit oxidative capacity and disrupt energy metabolism, potentially increasing the physiological importance of systemic aerobic capacity for supporting cerebral oxygen metabolism. Although the present study cannot establish mechanism, these findings may reflect improved oxygen delivery, enhanced cellular oxygen utilization, or a combination of both processes, and this framework may help explain the stronger association observed between CRF and OEF in participants with AD. In contrast, CH individuals may maintain sufficient oxygen delivery, uptake, and oxidation to meet metabolic demand, limiting the observable impact of CRF on OEF. Because CBF was lower in AD and strongly associated with OEF, it’s also possible that cerebral perfusion contributes to the observed relationship between CRF and OEF. Higher CRF may influence oxygen extraction indirectly through effects on cerebral perfusion, although the present study was not designed to formally evaluate mediation pathways. Future studies incorporating larger samples and mediation analyses are needed to determine whether CBF represents a mechanistic link between CRF and OEF.

Although we did not observe group differences in OEF, participants with AD exhibited lower resting CBF and GM, consistent with prior literature (Apostolova et al., 2007; Wu et al., 2021; Zhang H. et al., 2021; Zhang Q. et al., 2021). These findings suggest the presence of cerebrovascular dysfunction and neurodegeneration in our sample of AD. While mean arterial pressure, the primary driving force for cerebral perfusion, did not differ between groups, the reduced CBF observed in AD may reflect diminished cerebrovascular reserve, potentially increasing sensitivity to CRF.

Across the full cohort, higher CBF was strongly associated with lower OEF, consistent with the physiological relationship between cerebral oxygen delivery and oxygen extraction (Jiang et al., 2020b; Chong Chie et al., 2025; Herculano-Houzel and Rothman, 2022). Greater cerebral perfusion increases oxygen availability to brain tissue, reducing the fraction of delivered oxygen that must be extracted to satisfy resting metabolic demand. The absence of a significant CBF × diagnosis interaction suggests that this fundamental physiological relationship is preserved in both CH and AD despite differences in absolute cerebral perfusion. Whether this preserved resting relationship extends to dynamic cerebrovascular-metabolic responses during physiological stress remains unknown and warrants further investigation.

Regional CBF analyses further demonstrated that this relationship was not limited to global measures. Significant negative associations between CBF and OEF were observed across multiple regions involved in cognitive and autonomic regulation, including anterior cingulate cortex, insula, thalamus, medial frontal cortex, posterior cingulate cortex, and precuneus. Notably, several of these regions are components of the default mode network and are particularly vulnerable to AD-related metabolic dysfunction and hypoperfusion. These findings suggest that the coupling between cerebral perfusion and oxygen extraction is maintained across functionally important brain networks and may represent a fundamental aspect of neurovascular-metabolic regulation.

Although participants with AD exhibited significantly lower resting CBF, OEF did not differ between diagnostic groups. This finding suggests that resting cerebral oxygen extraction remains relatively preserved despite reductions in perfusion. One possible explanation is that compensatory increases in fractional oxygen extraction help maintain cerebral oxygen metabolism when oxygen delivery declines. Such compensation has been described in conditions of impaired cerebral perfusion and may represent an adaptive response that preserves resting metabolic function until vascular reserve becomes exhausted (Govindpani et al., 2019). Alternatively, the absence of elevated OEF despite lower CBF may indicate that compensatory oxygen extraction is insufficient to fully offset reductions in perfusion, resulting in lower cerebral metabolic rate of oxygen consumption. This interpretation is consistent with well-established cerebral hypometabolism observed in AD and may reflect limitations in vascular reserve, oxygen extraction capacity, cellular utilization of oxygen or a combination of these factors. Consequently, OEF should be interpreted together with measures of cerebral perfusion rather than in isolation when evaluating cerebrovascular physiology in aging and AD.

These findings may also have increased clinical relevance as vascular contributions to AD progression receive greater attention. Cardiovascular disease becomes more prevalent with advancing age (North and Sinclair, 2012), and frequently co-occurs with AD pathology, contributing to cerebrovascular dysfunction (Attems and Jellinger, 2014; Cortes-Canteli and Iadecola, 2020; Eisenmenger et al., 2023). More recently, these interactions have gained more attention with the emergence of anti-amyloid therapies, which are associated with treatment-related adverse events such as vasogenic edema and microhemorrhage (Chundu et al., 2025). Collectively, these findings suggest that CRF, CBF, and OEF provide complementary physiological information across different levels of the oxygen transport pathway. While CRF reflects systemic oxygen delivery capacity, CBF reflects cerebral oxygen delivery, and OEF reflects the fractional extraction required to maintain cerebral metabolism. Integrating these measures may therefore improve our understanding of cerebrovascular physiology and identify early physiological alterations associated with AD.

## 5 Limitations

Several limitations should be considered when interpreting these findings. Fist, the present study includes a relatively modest sample size. Although significant associations between CRF and OEF were observed in AD, the study may have been underpowered to detect smaller between-group differences in physiological measures. Replication in larger cohorts will be important to confirm the robustness and generalizability of these findings. Second, CBF was quantified using a single-delay pCASL acquisition. While this approach is widely used, it does not directly account for individual or regional differences in arterial transit time, which may influence CBF estimation. Future studies employing multi-delay ASL may help further characterize the contribution of transit time to observed perfusion differenced. Additionally, the CH group included several highly fit older adults. Therefore, the observed relationships may not generalize to less-fit older population.

## 6 Conclusion

Overall, these findings demonstrate that the relationship between cardiorespiratory fitness and cerebral oxygen extraction differs by diagnostic group, with higher CRF associated with reduced reliance on oxygen extraction in individuals with AD whereas the association did not reach statistical significance in CH. In contrast, the inverse relationship between cerebral blood flow and OEF was preserved across both groups, highlighting a fundamental physiological coupling between cerebral oxygen delivery and oxygen extraction. Together, these findings suggest that systemic fitness, cerebral perfusion, and oxygen extraction provide complementary information regarding cerebral oxygen metabolism. The observed associations between CRF and OEF appears to be most pronounced in individuals with AD, highlighting a potential disease-related physiological coupling that warrants further investigation.

## Data Availability

The raw data supporting the conclusions of this article will be made available by the authors, without undue reservation.
